# Indocyanine green fluorescence imaging ensures perfusion of the remnant stomach during laparoscopic splenectomy in a patient after distal gastrectomy: A case report

**DOI:** 10.1016/j.ijscr.2021.106111

**Published:** 2021-06-15

**Authors:** Hiroki Fujino, Minoru Nagayama, Yasutoshi Kimura, Masafumi Imamura, Takayuki Nobuoka, Ichiro Takemasa

**Affiliations:** Department of Surgery, Surgical Oncology and Science, Sapporo Medical University School of Medicine, 291 South 1, West 16, Chuo-ku, Sapporo, Hokkaido 060-8543, Japan

**Keywords:** Indocyanine green, Laparoscopic splenectomy, Distal gastrectomy, Remnant stomach

## Abstract

**Background:**

After distal gastrectomy, ischemic necrosis of the remnant stomach is a rare but serious complication. For distal pancreatectomy or splenectomy, ensuring adequate blood supply to the remnant stomach is important for patients with a history of distal gastrectomy. We report a case of successful splenectomy with indocyanine green (ICG) used to evaluate the blood supply to the remnant stomach in a patient after distal gastrectomy.

**Case presentation:**

A 65-year-old woman who underwent distal gastrectomy for gastric cancer a year earlier had a splenic tumor that was increasing in size. We planned laparoscopic splenectomy because there was a possibility of a malignant splenic tumor. Intraoperative ICG fluorescence imaging confirmed perfusion of the remnant stomach. The patient was discharged on postoperative day 8 after an uncomplicated postoperative course.

**Conclusion:**

ICG fluorescence imaging is useful for evaluating blood flow to the remnant stomach during laparoscopic splenectomy in patients after distal gastrectomy.

## Highlights

• Ischemic necrosis of the remnant stomach is a serious complication for patients with a history of distal gastrectomy.
• During laparoscopic surgery, the method for evaluating the blood supply to remnant stomach has not been established.
• ICG fluorescence imaging is only way to visually evaluate the blood supply to organs during surgery.
• Using ICG fluorescence imaging during laparoscopic splenectomy, ischemic necrosis of the remnant stomach can be prevented.

## 1 Introduction

After distal gastrectomy (DG), the blood supply to the remnant stomach comes from three sources: the (1) short gastric arteries (SGA), (2) posterior gastric artery (PGA), and (3) left inferior phrenic artery (LIPA). The SGA diverge from the splenic artery and the PGA may also branch off from the splenic artery. The LIPA arises from the aorta or celiac axis [1]. Ischemic necrosis of the remnant stomach after splenectomy is a serious complication that may occur in patients with a history of DG because the spleen and remnant stomach share arterial blood supply via the splenic artery and the SGA [2]. It was reported that in 28 cases of ischemic necrosis of the remnant stomach after DG, 16 cases of them (57%) had undergone distal gastrectomy and splenectomy simultaneously [2]. If perfusion of the remnant stomach is compromised, total gastrectomy should be considered during splenectomy.

If the fundic branches of the PGA and LIPA supply adequate blood flow during splenectomy, the remnant stomach could be preserved. Therefore, evaluation of remnant stomach perfusion is important to perform splenectomy safely. Herein we report successful splenectomy with remnant stomach preservation in a patient with a history of DG based on intraoperative evaluation of remnant stomach perfusion using laparoscopic indocyanine green (ICG) fluorescence imaging.

## 2 Case presentation

**Fig. 1:**
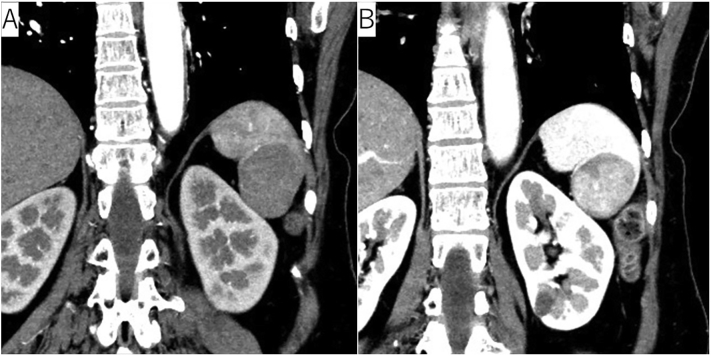
CT (A, arterial phase, coronal; B, portal phase, coronal); enhanced CT showed a tumor in the lower splenic pole with a diameter of 30 mm.

**Fig. 2:**
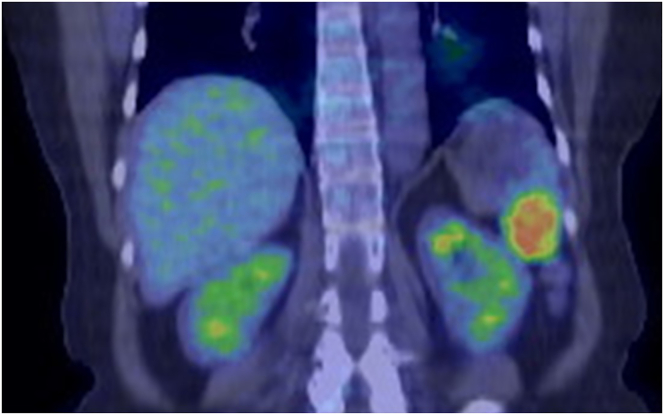
PET-CT showed abnormal FDG uptake (SUVmax, 7.5) in the splenic tumor.

**Fig. 3:**
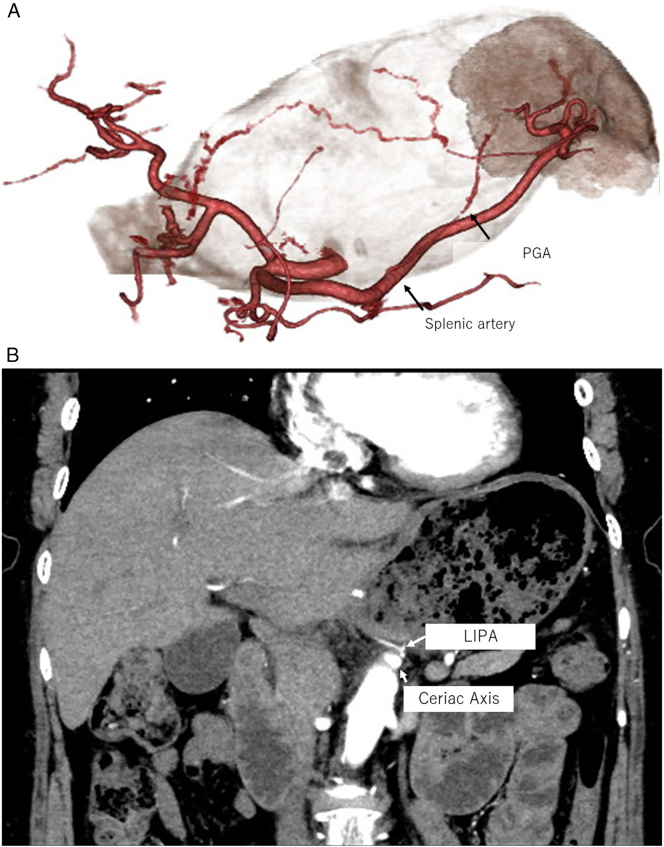
CT angiography; (A) three-dimensional CT imaging before splenectomy showed a developed PGA. (B) Arterial phase CT showed the LIPA branched from the celiac axis. LIPA, left inferior phrenic artery.

A 65-year-old woman with a history of laparoscopic DG with lymph node dissection for early gastric cancer 6 months earlier had a 40 mm splenic tumor that was growing. During laparoscopic DG, lymph node dissection around the common hepatic artery and ligation of the left gastric artery, right gastric artery, and left and right gastroepiploic arteries were performed. Pathological diagnosis of gastric cancer was pT1bN0M0 StageIA (UICC 8th TNM). The splenic tumor with 30 mm large in the lower pole of the spleen was incidentally detected before DG with enhanced computed tomography (CT). It was diagnosed as a benign tumor with well-defined margins and homogeneous enhancement (Fig. 1). The splenic tumor was observed for 6 months without resection after DG. Intraoperative biopsy of the splenic tumor was not performed during DG due to the risk of complications such as bleeding. However, in the subsequent enhanced CT suggested the tumor increased in size to a diameter of 40 mm and positron emission tomography showed abnormal ^18^F-fluorodeoxyglucose uptake in the splenic tumor (Fig. 2) with a possibility of being a malignant splenic tumor such as malignant lymphoma or metastasis. Three-dimensional CT angiography showed that the PGA and LIPA had become more developed to supply the remnant stomach (Fig. 3).

We planned to perform laparoscopic splenectomy without resection of the remnant stomach in case of adequate preservation of the blood supply to the remnant stomach after SGA resection. During laparoscopic splenectomy, we resected the SGA, so the arterial blood supply to the remnant stomach depended on the PGA and LIPA. We examined the blood supply to the remnant stomach with intraoperative ICG fluorescence imaging using a near-infrared camera system after clamping the splenic artery and vein at the splenic hilum, the planned transection line. First, 5 mg of ICG in 2 mL of saline was gently injected through a peripheral vein. ICG fluorescence imaging was illuminated with a near-infrared laser beam in the laparoscopic system. Imaging was generated using the 1588 AIM Endoscopic Near Infrared Visualization camera system (Stryker Corporation, Kalamazoo, MI, USA). Within 45 s after ICG injection, blood flow from the greater curvature spreading to the lesser curvature of the remnant stomach was visualized with ICG fluorescence (Additional File 1, Video 1/Fig. 4). The remnant stomach did not show any perfusion defects, suggesting adequate blood supply from the PGA and LIPA, allowing for splenectomy with remnant stomach preservation. Total operative time was 143 min. Intraoperative blood loss was 5 mL.

The final pathological diagnosis of the splenic tumor was sarcoidosis. The patient was discharged on postoperative day 8 without complications. Two years after surgery, gastric cancer and sarcoidosis have progressed without recurrence.

This work has been reported in line with the SCARE criteria [3].

## 3 Discussion

In patients with a history of DG, partial deficit of the blood supply to the remnant stomach can cause severe complications such as ischemic necrosis. Previous reports on ischemia of the remnant stomach after DG suggested that the splenic artery is essential for maintaining the blood supply to the remnant stomach [1,2,4,5] and simultaneous DG and splenectomy led to a high rate of ischemic necrosis of the proximal stomach and a mortality rate was as high as 70% [1,2]. Therefore, when splenectomy is planned for a patient with a history of DG, blood flow to the remnant stomach should be closely assessed.

Successful preservation of the remnant stomach in this case may be attributed to extragastric blood supply via the well-developed PGA and LIPA branches and a rich mucosal plexus arising from a plexus of large submucosal vessels. The intramural capillary networks, along with the extragastric arterial blood supply from the PGA and LIPA, could compensate for the loss of blood flow from the SGA after splenectomy.

Recent reports suggest that ICG fluorescence imaging could be used to assess blood supply to a variety of organs [[6], [7], [8], [9]]. Conventionally, intraoperative evaluation of blood flow to organs was based on the color tone of the organs wall and the presence or absence of bleeding and arterial pulsation. However, ICG fluorescence imaging, which observe the organs using a camera with near-infrared light after intravenous injection of ICG, can confirm blood flow to organs with fluorescence, clearly delineating the boundary between areas with good and poor perfusion [[8], [9], [10], [11]]. In our case, ICG fluorescence imaging was performed to visualize the blood flow and vascular distribution in the remnant stomach. Intraoperative ICG fluorescence imaging has been used to evaluate microvascular circulation of free flaps in reconstructive surgery. Blood flow to the remnant stomach could be visualized in real time with a near-infrared color camera system and a charge-coupled device camera to detect the near-infrared fluorescence signal emitted by intravenously injected ICG. It was reported that ICG fluorescence imaging might help to estimate the blood supply of visceral anastomosis in upper gastrointestinal surgery. A few retrospective studies have evaluated gastric conduit using ICG fluorescence imaging in esophagectomy, showing a number of changes in anastomotic leak rate from 14.8% to 1.7%, and 15% to 6%, respectively [8,12].

There are some limitations to detecting blood flow to hollow organs using ICG fluorescence imaging. Since ICG fluorescence imaging indicates only blood flow on the serosal surface, it cannot reflect blood flow in deeper parts of the tissue such as the mucosal surface. Another limitation is the challenge in objectively quantifying fluorescence from organs, for example the time from ICG injection to fluorescence the tissue or the strength of the color tone of the fluorescent tissue. Therefore, criteria for determining ischemia have not been established yet. ICG fluorescence imaging is a technique which is still under evaluation although it is used in the clinical. Further consideration and accumulation of cases to evaluate intestinal blood flow using ICG fluorescence imaging seems to be necessary.

## 4 Conclusion

Using ICG fluorescence imaging during laparoscopic splenectomy, we could evaluate the blood supply to the remnant stomach and preserve the remnant stomach safely in a patient with a history of DG. The patient had no ischemic complications after surgery. ICG fluorescence imaging is useful for evaluating blood flow to the remnant stomach.

The following is the supplementary data related to this article.

Additional File 1, Video 1/Fig. 4 During laparoscopic splenectomy, we resected the SGA and clamped the SPA/V before injecting ICG. Within a minute after ICG injection, blood flow was confirmed by ICG fluorescence from the greater curvature of the remnant stomach. (A; 45 s after injecting ICG/B; 60 s after injecting ICG). Additional File 1, Video 1/Fig. 4

## Abbreviations

CT: computed tomography
DG: distal gastrectomy
ICG: indocyanine green
LIPA: left inferior phrenic artery
PGA: posterior gastric artery
SGA: short gastric arteries

## Availability of data and materials

The data associated with this case report are available from the corresponding author, MN, upon appropriate request.

## Declaration of competing interest

The authors declare that they have no conflicts of interests.

## References

[1] Takahashi H., Nara S., Ohigashi H., Sakamoto Y., Gotoh K. (2013). Is preservation of the remnant stomach safe during distal pancreatectomy in patients who have undergone distal gastrectomy?. World J. Surg..

[2] Isabella V., Marotta E., Bianchi F. (1984). Ischemic necrosis of proximal gastric remnant following subtotal gastrectomy with splenectomy. J. Surg. Oncol..

[3] Agha R.A., Franchi T., Sohrabi C., Mathew G., for the SCARE Group (2020). The SCARE 2020 guideline: updating consensus Surgical CAse REport (SCARE) guidelines. Int. J. Surg..

[4] Hanaoka M., Shinohara H., Haruta S., Tate T., Fujii T. (2014). Successful distal gastrectomy after distal pancreatectomy combined with splenectomy by assuring the blood flow to the remnant stomach from the left inferior phrenic artery. Hepatogastroenterology.

[5] Sasako M. (1998). Risk factors for surgical treatment in the Dutch Gastric Cancer Trial. Br. J. Surg..

[6] Miyauchi W., Shishido Y., Kono Y., Murakami Y., Kuroda H. (2018). Less invasive surgery for remnant stomach cancer after esophago-proximal gastrectomy with ICG-guided blood flow evaluation. Yonago Acta Med.

[7] Maruoka S., Ojima T., Nakamori M., Nakamura M., Hayata K. (2017). Usefulness of indocyanine green fluorescence imaging: a case of laparoscopic distal gastrectomy after distal pancreatectomy with splenectomy. Surg. Endosc.

[8] Ohi M., Toiyama Y., Mohri Y., Saigusa S., Ichikawa T. (2017). Prevalence of anastomotic leak and the impact of indocyanine green fluorescein imaging for evaluating blood flow in the gastric conduit following esophageal cancer surgery. Esophagus.

[9] Shimada Y., Okumura T., Nagata T., Sawada S., Matsui K. (2011). Usefulness of blood supply visualization by indocyanine green fluorescence for reconstruction during esophagectomy. Esophagus.

[10] Ishii M., Hamabe A., Okita K. (2019). Efficacy of indoyanine green fluorescence angiography in preventing anastomotic leakage after laparoscopic colorectal cancer surgery. Int. J. Color. Dis..

[11] Murono S., Ishikawa N., Ohtake H., Tsuji A. (2014). Intraoperative free jejunum flap monitoring with indocyanine green near infrared angiography. Eur. Arch. Otorhinolaryngol.

[12] Kitagawa H., Namikawa T., Iwabu J., Fujisawa K. (2018). Assessment of the blood supply using the indocyanine green fluorescence method and postoperative endoscopic evaluation of anastomosis of the gastric tube during esophagectomy. Surg. Endosc..

